# Using focused pharmacovigilance for ensuring patient safety against antileishmanial drugs in Bangladesh’s National Kala-azar Elimination Programme

**DOI:** 10.1186/s40249-018-0461-0

**Published:** 2018-08-13

**Authors:** Md. Sakhawat Hossain, Amresh Kumar, A.F.M Akhtar Hossain, Md. Mahshin, Abhijit Sharma, Md. Akter Hossain, Varun Sharma, Rashidul Haque, A.K.M Shamsuzzaman, Shomik Maruf, Prakash Ghosh, Vivek Ahuja, Dinesh Mondal

**Affiliations:** 1 68 Shaheed Taj Uddin Ahmed Sarani, Parasitology Laboratory, International Centre for Diarrhoeal Disease Research (icddr,b), Mohakhali, Dhaka, 1212 Bangladesh; 2 Program for Appropriate Technology in Health (PATH), 15th Floor, Dr. Gopal Das Bhawan 28 Barakhamba Road, New Delhi, 110001 India; 3 Communicable Disease Control Unit, Directorate General of Health Services, Government of Bangladesh, Mohakhali, Dhaka, 1212 Bangladesh; 4 Adverse Drug Reaction Monitoring Cell, Directorate General of Drug Administration (DGDA), Government of Bangladesh, Mohakhali, Dhaka, 1212 Bangladesh; 5 Nutrition Infection Interaction Research Group, Nutrition and Clinical Services Division (icddr,b), Dhaka, 1212 Bangladesh; 6 Infectious Disease Research Institute (IDRI), Seattle, USA

**Keywords:** Visceral leishmaniasis, Pharmacovigilance, Antileishmanial drugs, Health system, Bangladesh

## Abstract

**Background:**

Adverse effects of antileishmanial drugs can affect patients’ quality of life and adherence to therapy for visceral leishmaniasis (VL) and post-kala-azar dermal leishmaniasis (PKDL). In Bangladesh, there are 26 treatment centers that manage leishmaniasis cases coming from 100 endemic upazilas (subdistricts) of 26 districts (these include VL, PKDL, treatment failure, and relapse VL and cutaneous leishmaniasis cases). This study aimed to investigate the feasibility of using focused pharmacovigilance for VL (VLPV) in Bangladesh’s National Kala-azar Elimination Programme for the early detection and prevention of expected and unexpected adverse drug reactions (ADRs).

**Methods:**

This activity has been going on since December 2014. Activity area includes secondary public hospital or Upazila health complex (UHC) in hundred sub districts and Surya Kanta Kala-azar Research Center (SKKRC) in Mymensingh District, a specialized center for management of complicated VL and PKDL cases. Communicable Disease Control (CDC) of the Directorate General of Health Services (DGHS) assigned twenty five of hundred UHCs and SKKRC (total 26) as treatment centers depending on their suitable geographical location. This was implemented for better management of VL cases with Liposomal Amphotericin B (AmBisome®) to ensure patient convenience and proper utilization of this expensive donated drug. A VLPV expert committee and a UHC VLPV team were established, an operational manual and pharmacovigilance report forms were developed, training and refresher training of health personnel took place at UHCs and at the central level, collected information such as patient data including demographics, treatment history and response, adverse events were analyzed. This report includes information for the period from December 2014 to December 2016.

**Results:**

From December 2014 to December 2016, 1327 leishmaniasis patients were treated and 1066 (80%) were available for VLPV. Out of these, 57, 33, 9, and 1% were new VL, PKDL, VL relapse, and other cases, respectively. Liposomal amphotericin B was mostly used (82%) for case management, followed by miltefosine (20%) and paromomycin (3%). Out of the 1066 patients, 26% experienced ADRs. The most frequent ADR was fever (17%, 176/1066), followed by vomiting (5%, 51/1066). Thirteen serious adverse events (SAEs) (eight deaths and five unexpected SAEs) were observed. The expert committee assessed that three of the deaths and all unexpected SAEs were possibly related to treatment. Out of the five unexpected SAEs, four were miltefosine-induced ophthalmic complications and the other was an AmBisome®-induced avascular necrosis of the nasal alae. The Directorate General of the Drug Administration entered the ADRs into the World Health Organization Uppsala Monitoring Centre (WHO-UMC) VigiFlow database.

**Conclusions:**

This study found that VLPV through NKEP is feasible and should be continued as a routine activity into the public health system of Bangladesh to ensure patient safety against anti-leishmanial drugs.

**Electronic supplementary material:**

The online version of this article (10.1186/s40249-018-0461-0) contains supplementary material, which is available to authorized users.

## Multilingual abstracts

Please see Additional file 1 for translations of the abstract into the five official working languages of the United Nations.

## Background

Visceral leishmaniasis (VL), also called kala-azar, is a vector-borne anthroponotic infection caused by the protozoan *Leishmania donovani*, which is transmitted by the bite of an infected female *Phlebotomine argentipes* (sandfly). VL is a deadly disease if it is not treated properly, and is a public health problem in the Indian subcontinent (ISC) and East Africa [1, 2]. Even with treatment, the case fatality rate ranges from 1 to 10% in different VL-endemic countries [2, 3]. India, Bangladesh, and Nepal together contributed to 60% of the world’s VL burden until 2006. In the ISC, the disease is now at a controllable state due to the availability of effective drugs and diagnostics [4].

The VL elimination initiative in the ISC started in 2005 when the reported number of cases were about 10 000, 40 000 and 3000 respectively in Bangladesh, India and Nepal [4]. By 2016, a dramatic reduction of the VL burden (a decrease of almost 10 times) in the member countries occurred [1, 5]. Bangladesh and Nepal have already achieved the elimination target of keeping VL incidence less than one per ten thousand people at Upazila and District level in Bangladesh and Nepal respectively. Whereas, India is very close to achieving it [5]. An important contributor to the success of the elimination program is the availability of antileishmanial drugs for the treatment of VL and post-kala-azar dermal leishmaniasis (PKDL) [6]. For more than 70 years, sodium stibogluconate was the only drug for treating VL, but it has been phased out in 2013 in the ISC due to its toxicity and reduced efficacy [2]. Fortunately, liposomal amphotericin B (L-AmB, AmBisome®), miltefosine (MF), and paromomycin (PM) are successfully being used in monotherapy or in combination for the treatment of VL [7–10]. Single-dose L-AmB is now the first choice for new VL treatment in Bangladesh following the successful implementation of single dose liposomal amphotericin B (SDA) in Upazila health complex (UHC) through the public health system [11, 12]. Combination therapy is recommended when single dose L-AmB therapy cannot be applied [12]. MF monotherapy is not encouraged for the treatment of VL due to its less efficacy, longer duration of treatment. Hence inadequate treatment compliance and high relapse rate after cure. However, it remains the first treatment regimen for PKDL, a dermatological sequelae of the *L. donovani* infection [2, 12]. Currently L-AmB, PM, MF, and amphotericin B deoxycholate (ABD) are the drugs approved by the Government of Bangladesh (GoB) for the management of VL and PKDL patients [12]. As none of the above drugs have been initially formulated for leishmaniasis treatment, these drugs are not licensed by the Food and Drug Administration for antileishmanial treatment [13]. However, considering their safety, efficacy, and availability, these drugs are recommended for use by the World Health Organization (WHO), as well as by VL experts.

**Fig. 1:**
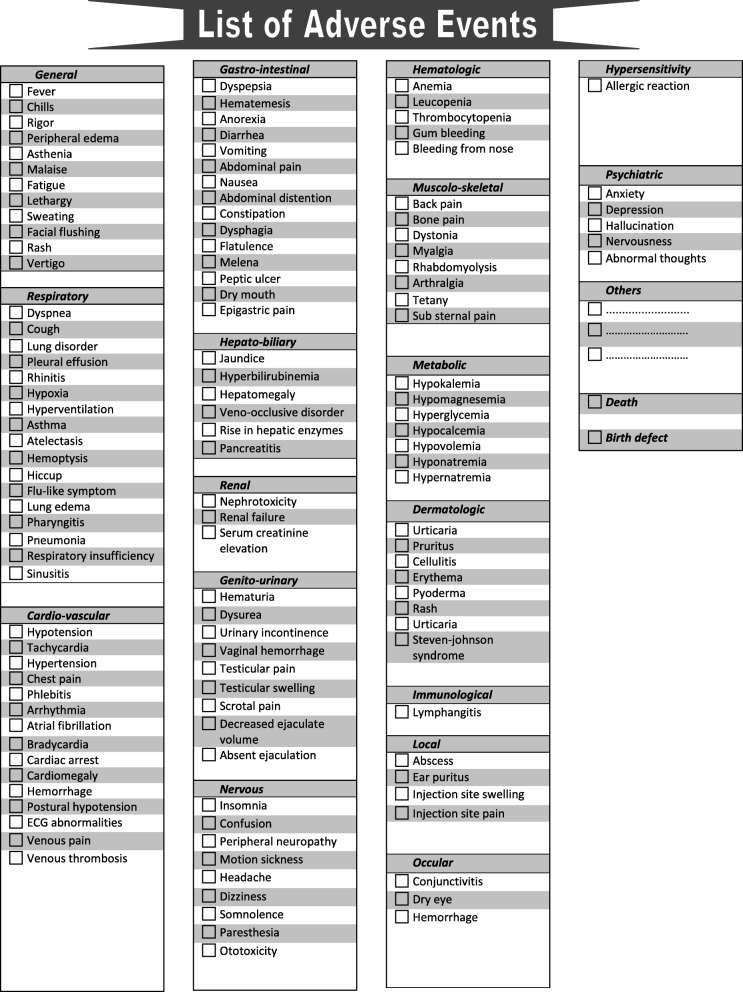
List of expected adverse drug reactions

Like other drugs, antileishmanial drugs have side effects or adverse drug reactions (ADRs) (see Fig. 1). Adverse effects due to drugs can significantly affect the quality of life and adherence to therapy. Acute hepatitis, renal failure, ototoxicity, and even deaths have been reported during treatment of VL [8, 10, 14]. Therefore monitoring ADRs to ensure patients’ wellbeing and safety is imperative [15].

The term “pharmacovigilance” has been coined to define the science and activity relating to the detection, assessment, understanding, and prevention of ADRs or other possible drug-related problems [16]. In Bangladesh, pharmacovigilance was introduced in 1999, but effective functioning started in 2013 when the Adverse Drug Reaction Monitoring (ADRM) Cell was established within the Directorate General of Drug Administration (DGDA), and designated as the National Drug Monitoring Center by the Ministry of Health and Family Welfare [17]. After its establishment, the ADRM Cell had no capacity for monitoring ADRs related to antileishmanial drugs because of the lack of human resources and no experience about pharmacovigilance of the drugs which are not available in private market. Upon recommendation of the regional technical advisory group for VL elimination as well as international VL experts, Bangladesh’s National Kala-azar Elimination Programme (NKEP) introduced focused pharmacovigilance for monitoring ADRs of antileishmanial drugs to enhance patients’ care [18, 19]. Current pharmacovigilance for VL (VLPV) activity is the brainchild of the NKEP, and is believed to be an effective model of pharmacovigilance to ensure maximum therapeutic benefits for VL/PKDL patients through the early detection and prevention of ADRs.

The objective of this study was to investigate the feasibility of VLPV in Bangladesh’s public health system to enhance patient care and ensure patient safety during and after treatment with currently available antileishmanial drugs. The VLPV is ongoing, and here we report our observation for the first 2 years (December 2014 to December 2016) of this important activity of the NKEP.

## Methods

### VLPV sites

About 100 upazilas (subdistricts) of 26 districts are considered as endemic for VL in Bangladesh. This activity has been going on since December 2014. Activity area includes secondary public hospital or Upazila health complex (UHC) in hundred sub districts and Surya Kanta Kala-azar Research Center (SKKRC) in Mymensingh District which is the only specialized center for management of complicated VL and PKDL, treatment failure, relapse VL and cutaneous leishmaniasis (CL) cases of all ages and sexes, including pregnant women. Communicable Disease Control (CDC) of the Directorate General of Health Services (DGHS) assigned twenty five of hundred UHCs and SKKRC (total 26) as treatment centers depending on their suitable geographical location. This was implemented for better management of VL cases with Liposomal Amphotericin B to ensure patient convenience and proper utilization of this expensive donated drug. The 26 treatment centers are the sites for VL treatment in Bangladesh while PKDL treatment with MF is available in all hundred UHCs and SKKRC.

### VLPV methods

**Fig. 2:**
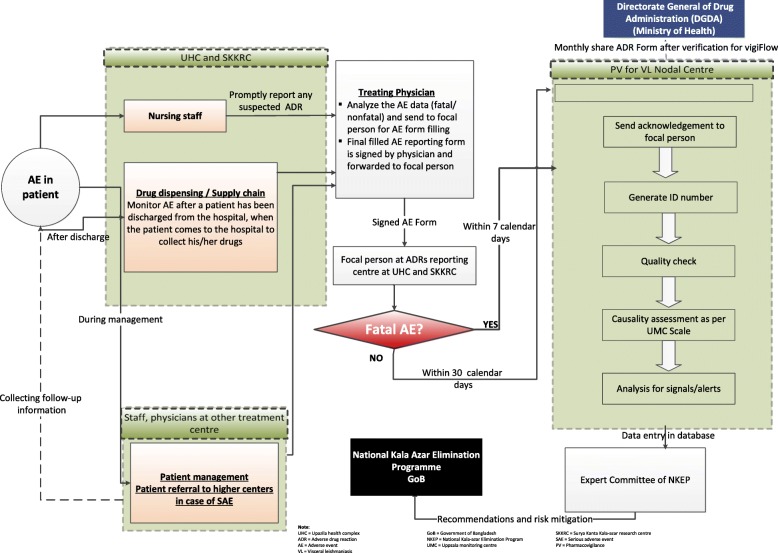
Activity framework for the operational manual for pharmacovigilance for visceral leishmaniasis in Bangladesh

1. ***Formation of the VLPV expert committee:*** The VLPV expert committee comprises 10 members from different governmental, non-governmental, and foreign organizations, including the Directorate General of Health Services (DGHS); the ADRM Cell of the DGDA; the Dhaka Medical College; the International Centre for Diarrhoeal Disease Research, Bangladesh (icddr,b); and Program for Appropriate Technology in Health (PATH). The members include the program manager of kala-azar at the Center for Disease Control (CDC), two expert pharmacologists, two representatives from the DGDA, two senior physicians with experience in VL management, two international VL experts, and the director of the CDC, DGHS, who is also the committee’s chair. Meetings are held on a half-yearly basis, however, there is provision for holding an emergency meeting, if required. The objectives of the committee are to supervise VLPV activities and conduct causality assessments of both expected and unexpected ADRs related to antileishmanial drugs.
2. ***Operational manual for focused pharmacovigilance:*** The expert committee developed the activity framework (see Fig. 2) and operational manual (see Additional file 2) for focused VLPV in Bangladesh. The experts on pharmacovigilance and VL participated in the preparation of the manual (Additional file 2). It includes the purpose, procedures, and roles and responsibilities of pharmacovigilance teams (a medical officer, staff nurse, and statistician) at 100 UHCs, as well as of the person responsible for this at the NKEP, a focal person at the ADRM Cell, and one at the icddr,b. The manual also describes what information needs to be collected regarding pharmacovigilance, how to generate reports, the reporting procedures, frequency (monthly) and timeline (within 10th of next month and in case of SAEs it is within 7 days). Pharmacovigilance reporting forms were based on the existing pharmacovigilance reporting formats of the ADRM Cell with modifications made to account for VLPV (see Additional file 2). The operational manual also includes definitions of ADRs and serious adverse events (SAEs), an ADR severity assessment tool, the WHO-Uppsala Monitoring Centre (WHO-UMC) causality assessment criteria and structure, and the role of the VLPV expert committee [20]. Based on the available literature, a list of expected ADRs to antileishmanial drugs was also developed (see Fig. 1). All treatment centers involved in the program have been instructed to report ADRs monthly, within 10th of next month in case of SAEs it is within 7 days to the NKEP.
3. ***Training on focused pharmacovigilance for VL:*** National and international experts trained each pharmacovigilance team along with NKEP program personnel at the central level on ADR assessment, pharmacovigilance reporting, and report analysis to ensure the quality of the reporting system.
4. ***Case identification and surveillance:*** The following activities are conducted for case identification and pharmacovigilance surveillance:
  4.1 *Monitoring of hospital patients:* Trained medical doctors and nurses monitor VL patients in 26 treatment centers during their treatment in hospitals, and their pharmacovigilance reports are generated upon discharge. Pharmacovigilance reports of the PKDL patients are generated after completion of treatment or during the occurrence of ADRs. PKDL patients who need intravenous infusion with L-AmB/ABD are treated and monitored in SKKRC.
  4.2 *Active surveillance:* The NKEP has planned 5 years of follow-up of treated VL and PKDL patients. The follow-up visit schedule for the treated patients includes a visit at 1, 6, and 12 months following treatment and then every 6 months for the next 4 years.
  4.3 *Passive surveillance:* Passive surveillance includes noting the number of VL and PKDL patients treated at subdistrict hospitals and reporting it to the central level of the NKEP.
  4.4 *Deaths:* If a treated VL or PKDL patient dies, a medical doctor from the treatment center carries out a verbal autopsy via a home visit. If the death occurred at a health facility, then document analysis is carried out by an expert from the SKKRC to identify the cause of death. An autopsy is not accepted culturally in Bangladesh and is done only in the case of a legal issue.
5. ***Reporting:*** The VLPV team in the subdistrict hospital/SKKRC sends completed VLPV forms to the NKEP, CDC, DGHS. The central level verifies the collected information with the treatment center when needed. The final forms are sent to the ADRM Cell, which inputs the ADRs into the WHO VigiFlow database. VigiFlow is an individual case safety report management web-based system. It is developed and hosted by the UMC. A copy of the report is also sent to the icddr,b VLPV team for data analysis, report preparation, and presentation to the NKEP and other stakeholders.
6. ***Data analysis:*** A data entry program based on VLPV reporting forms was developed. For this study, all patient data including demographics, treatment history, and adverse events were entered using individual patient IDs and initials (see Additional file 2). A descriptive statistical analysis was done to determine the frequency of expected and unexpected drug reactions to antileishmanial drugs including L-AmB monotherapy, MF monotherapy, PM monotherapy, and combination therapy (L-AmB + MF, L-AmB + PM, MF + PM). Comparison of proportions was done using the chi-square test. All analyses were done using SPSS version 20 (IBM Corporation, Armonk, NY, USA).
7. ***Ethical considerations:*** This study was approved by the CDC, DGHS, GoB DGDA, GoB, and the research administration of icddr,b. All patient-related data were retrieved upon the consent of the CDC, GoB. As the CDC has the authority to provide patient-related data to any research body for passive data analysis, individual patient consent was not required to avail treatment-related data. Further, according to the NKEP, verbal autopsy is mandatory for VL treatment-related death cases. Therefore, the NKEP maintains ethical procedures to collect data through verbal autopsy. As the treatment centers included in this study fall under the CDC, DGHS, GoB, consent from the CDC was enough for including all treatment centers. All participating institutes signed a joint memorandum of understanding before initiating the activity.

## Results

### Feasibility of VLPV and case management

After the commencement of the VLPV activity in September 2014, only 10 out of 25 UHCs and SKKRC with the VL treatment facility had reported ADRs after the first training was conducted. Rest of the 74 UHCs did not report as they were not designated VL treatment centers during those period. The number of UHCs that reported ADRs went up to 13 (50%), 23 (88%), and 26 (100%) after the first, second, and third refresher trainings, respectively.

According to disease surveillance figures from the CDC at the DGHS, there were a total of 1327 leishmaniasis patients from December 2014 to December 2016. Of these, 807 (61%) were VL, 399 (30%) were PKDL, 111 (8%) were VL relapse, and 14 (1%) were other cases (four were treatment failures, two were CL, and eight were para-KDL, a condition when VL and PKDL exist simultaneously in the same patient). The 26 VL treatment centers sent 1066 VLPV forms, indicating that 80% of 1327 patients were under VLPV *(see* Table 1*)*. VLPV forms for rest of the 261 patients were not available. Of the 1066 cases, the numbers of new VL, PKDL, VL relapse, and other cases were 608 (57%), 351 (33%), 93 (9%), and 14 (1%), respectively. The vast majority (95%; 1011/1066) had been treated with monotherapy and 5% (55/1066) received combination therapy.

**Table 1:** Patients’ distribution based on treatment options along with adverse drug reactions

	L-AMB (ADR: *n*; percentage)	MILTE (ADR: *n*; percentage)	PARO (ADR: *n*; percentage)	L-AMB & MILTE (ADR: *n*; percentage)	L-AMB & PARO (ADR: *n*; percentage)	MILTE &PARO (ADR: *n*; percentage)	Total (ADR: *n*; percentage)
NKA	585 (179; 31%)	12 (2; 2/17)	0 (0; 0)	1 (0; 0)	9 (0; 0)	1 (0; 0)	608 (181; 30%)
PKDL	155 (41; 26%)	181 (29; 16%)	0 (0; 0)	15 (3; 3/15)	0 (0; 0)	0 (0; 0)	351 (73; 21%)
RELAPSE VL	64 (17; 27%)	0 (0; 0)	1 (0; 0)	7 (0; 0)	21 (0; 0)	0 (0; 0)	93 (17; 18%)
KATF	3 (2; 2/3)	0 (0; 0)	0 (0; 0)	1 (0; 0)	0 (0; 0)	0 (0; 0)	4 (2; 2/4)
RELAPSE AND PKDL	5 (2; 2/5)	0 (0; 0)	0 (0; 0)	0 (0; 0)	0 (0; 0)	0 (0; 0)	5 (2; 2/5)
CL	2 (1; 1/2)	0 (0; 0)	0 (0; 0)	0 (0; 0)	0 (0; 0)	0 (0; 0)	2 (1; 1/2)
NKA WITH PKDL	3 (0; 0)	0 (0; 0)	0 (0; 0)	0 (0; 0)	0 (0; 0)	0 (0; 0)	3 (0; 0)
Total (ADR: *n*; percentage)	817 (242; 30%)	193 (31; 16%)	1 (0; 0)	24 (3; 13%)	30 (0; 0)	1 (0; 0)	1066 (276; 26%)^a^

Note:

*ADR* adverse drug reactions, *CL* cutaneous leishmaniasis, *KATF* Kala-azar treatment failure, *L-AMB* liposomal amphotericin B, *MILTE* miltefosine, *NKA* New Kala-azar, *PARO* paromomycin, *PKDL* Post Kala-azar dermal leishmaniasis, *VL* visceral leishmaniasis

^**a**^Where the percentage value has been calculated from the total number of ADRs, irrespective of disease type and treatment method

Of these 1066 patients, the majority (82%; 871/1066) received L-AmB either in mono or combination therapy. L-AmB was deployed mostly in monotherapy (94%, 817/871) for treatment of 96% (585/608) of new VL cases, 44% (155/351) of PKDL cases, 69% (64/93) of VL relapse cases, and 93% (13/14) of the other cases. L-AmB was used to manage 5% (54/1066) of the cases in combination with either MF or PM. These regimens containing L-AmB were mostly used to treat VL relapse (30%, 28/93), followed by PKDL (5%, 16/351) and new VL (1.6%, 10/608) cases. Overall, 20% (218/1066) of patients received MF. Mostly MF was administered in monotherapy (89%, 193/218) to treat 52% (181/351) of PKDL cases and 2% (12/608) of new VL cases. The remaining 11% of patients were administered MF in combination with PM/L-AmB. In combination therapy, MF was mostly used for VL relapse cases (7.5%, 7/93), followed by PKDL cases (4%, 15/351) and new VL cases (0.3%, 2/608). PM was used to manage 3% of the patients (32/1066). It was used mostly (97%, 31/32) in combination with L-AmB (*n* = 30) and MF (*n* = 1) to treat 23% of VL relapse cases (21/93) and 1.4% of new VL cases (9/608). Only one case of VL relapse (1%, 1/93) received PM monotherapy. None of the patients received non-L-AmB or sodium stibogluconate.

### Expected ADRs

Forty-two types of ADRs were found, but only nine had a frequency of more than 1% (see Table 2). About a quarter (26%; 276/1066) of patients experienced ADRs, which was not found to be significantly associated with the sex or age range of patients. The most frequent ADR was fever (17%; 176/1066), followed by vomiting (5%; 51/1066), chills (4%; 42/1066), and rigor (3%; 33/1066).

**Table 2:** Observed frequency of expected adverse reactions to anti-leishmanial drugs

Serial No.	Adverse reaction	Frequency % (*n*/*N*)	Liposomal amphotericin B (L-AmB) mono therapy (*n*/*N*)	Miltefosine mono therapy (*n*/*N*)	Paromomycin mono therapy (*n*/*N*)	L-AmB and miltefosine combination therapy (*n*/*N*)	L-AmB and paromomycin combination therapy (*n*/*N*)	Miltefosine and paromomycin combination therapy (*n*/*N*)
1.	Fever	16.5 (176/1066)	20.4 (167/817)	4.7 (9/193)	0 (0/1)	0 (0/24)	0 (0/30)	0 (0/1)
2.	Vomiting	4.8 (51/1066)	4.2 (34/817)	8.8 (17/193)	0 (0/1)	0 (0/24)	0 (0/30)	0 (0/1)
3.	Chills	3.9 (42/1066)	4.9 (40/817)	0.5 (1/193)	0 (0/1)	4.2 (1/24)	0 (0/30)	0 (0/1)
4.	Rigor	3.1 (33/1066)	3.9 (32/817)	0 (0/193)	0 (0/1)	4.2 (1/24)	0 (0/30)	0 (0/1)
5.	Nausea	2.2 (23/1066)	1.5 (12/817)	5.7 (11/193)	0 (0/1)	0 (0/24)	0 (0/30)	0 (0/1)
6.	Diarrhea	1.4 (15/1066)	1.8 (15/817)	0 (0/193)	0 (0/1)	0 (0/24)	0 (0/30)	0 (0/1)
7.	Facial flushing	1.4 (15/1066)	1.7 (14/817)	0 (0/193)	0 (0/1)	4.2 (1/24)	0 (0/30)	0 (0/1)
8.	Back pain	1.2 (13/1066)	1.5 (12/817)	0 (0/193)	0 (0/1)	4.2 (1/24)	0 (0/30)	0 (0/1)
9.	Anorexia	1.0 (11/1066)	0.4 (3/817)	4.1 (8/193)	0 (0/1)	0 (0/24)	0 (0/30)	0 (0/1)
10.	Chest pain	0.8 (9/1066)	1.0 (8/817)	0 (0/193)	0 (0/1)	4.2 (1/24)	0 (0/30)	0 (0/1)
11.	Abdominal pain	0.8 (8/1066)	0.7 (6/817)	1.0 (2/193)	0 (0/1)	0 (0/24)	0 (0/30)	0 (0/1)
12.	Headache	0.8 (8/1066)	0.5 (4/817)	2.1 (4/193)	0 (0/1)	0 (0/24)	0 (0/30)	0 (0/1)
13.	Death	0.8 (8/1066)	1.0 (8/817)	0 (0/193)	0 (0/1)	0 (0/24)	0 (0/30)	0 (0/1)
14.	Heaviness of head	0.7 (7/1066)	0.9 (7/817)	0 (0/193)	0 (0/1)	0 (0/24)	0 (0/30)	0 (0/1)
15.	Rash	0.7 (7/1066)	0.9 (7/817)	0 (0/193)	0 (0/1)	0 (0/24)	0 (0/30)	0 (0/1)
16.	Sweating	0.6 (6/1066)	0.6 (5/817)	0 (0/193)	0 (0/1)	4.2 (1/24)	0 (0/30)	0 (0/1)
17.	Malaise	0.6 (6/1066)	0 (0/817)	3.1 (6/193)	0 (0/1)	0 (0/24)	0 (0/30)	0 (0/1)
18.	Vertigo	0.4 (4/1066)	0.5 (4/817)	0 (0/193)	0 (0/1)	0 (0/24)	0 (0/30)	0 (0/1)
19.	Hypotension	0.4 (4/1066)	0.5 (4/817)	0 (0/193)	0 (0/1)	0 (0/24)	0 (0/30)	0 (0/1)
20.	Tachycardia	0.4 (4/1066)	0.4 (3/817)	0 (0/193)	0 (0/1)	4.2 (1/24)	0 (0/30)	0 (0/1)
21.	Dyspnea	0.4 (4/1066)	0.4 (4/817)	0 (0/193)	0 (0/1)	0 (0/24)	0 (0/30)	0 (0/1)
22.	Chest tightness	0.3 (3/1066)	0.4 (3/817)	0 (0/193)	0 (0/1)	0 (0/24)	0 (0/30)	0 (0/1)
23.	Weakness	0.3 (3/1066)	0.4 (3/817)	0 (0/193)	0 (0/1)	0 (0/24)	0 (0/30)	0 (0/1)
24.	Cough	0.1 (1/1066)	0 (0/817)	0 (0/193)	0 (0/1)	4.2 (1/24)	0 (0/30)	0 (0/1)
25.	Dyspepsia	0.2 (2/1066)	0.2 (2/817)	0 (0/193)	0 (0/1)	0 (0/24)	0 (0/30)	0 (0/1)
26.	Hypertension	0.1 (1/1066)	0.1 (1/817)	0 (0/193)	0 (0/1)	0 (0/24)	0 (0/30)	0 (0/1)
27.	Jaundice	0.1 (1/1066)	0.1 (1/817)	0 (0/193)	0 (0/1)	0 (0/24)	0 (0/30)	0 (0/1)
28.	Leucopenia	0.1 (1/1066)	0.1 (1/817)	0 (0/193)	0 (0/1)	0 (0/24)	0 (0/30)	0 (0/1)
29.	Bone pain	0.1 (1/1066)	0.1 (1/817)	0 (0/193)	0 (0/1)	0 (0/24)	0 (0/30)	0 (0/1)
30.	Arthralgia	0.1 (1/1066)	0.1 (1/817)	0 (0/193)	0 (0/1)	0 (0/24)	0 (0/30)	0 (0/1)
31.	Allergic reaction	0.1 (1/1066)	0.1 (1/817)	0 (0/193)	0 (0/1)	0 (0/24)	0 (0/30)	0 (0/1)
32.	Belching	0.1 (1/1066)	0.1 (1/817)	0 (0/193)	0 (0/1)	0 (0/24)	0 (0/30)	0 (0/1)
33.	Injection site swelling	0.1 (1/1066)	0.1 (1/817)	0 (0/193)	0 (0/1)	0 (0/24)	0 (0/30)	0 (0/1)
34.	Lymphadenopathy	0.1 (1/1066)	1 (1/817)	0 (0/193)	0 (0/1)	0 (0/24)	0 (0/30)	0 (0/1)
35.	Salivation	0.1 (1/1066)	0.1 (1/817)	0 (0/193)	0 (0/1)	0 (0/24)	0 (0/30)	0 (0/1)
36.	Respiratory insufficiency	0.1 (1/1066)	0.1 (1/817)	0 (0/193)	0 (0/1)	0 (0/24)	0 (0/30)	0 (0/1)
37.	Pruritus	0.1 (1/1066)	0.1 (1/817)	0 (0/193)	0 (0/1)	0 (0/24)	0 (0/30)	0 (0/1)
38.	Leucopenia	0.1 (1/1066)	0.1 (1/817)	0 (0/193)	0 (0/1)	0 (0/24)	0 (0/30)	0 (0/1)
39.	Annular corneal ulcer	0.1 (1/1066)	0 (0/817)	0.5 (1/193)	0 (0/1)	0 (0/24)	0 (0/30)	0 (0/1)
40.	Mooren’s ulcer	0.1 (1/1066)	0 (0/817)	0.5 (1/193)	0 (0/1)	0 (0/24)	0 (0/30)	0 (0/1)
41.	Avascular necrosis of alae of nose	0.1 (1/1066)	0.1 (1/817)	0 (0/193)	0 (0/1)	0 (0/24)	0 (0/30)	0 (0/1)
42.	Cervical lymphonode	0.1 (1/1066)	0 (0/817)	0.5 (1/193)	0 (0/1)	0 (0/24)	0 (0/30)	0 (0/1)

One hundred and seventy-six patients developed fever during monotherapy and combo therapy with L-AmB, of which 138 were new VL, 23 were PKDL, 12 were VL relapse, and 3 were other cases. Fever was more common (20%) among patients who received L-AmB. MF treatment aroused the same gastrointestinal (GI) symptoms, including vomiting, nausea, and anorexia. However, vomiting was more common during treatment with MF.

Chills were mostly observed in patients (42/1,066) receiving treatment with L-AmB (new VL [*n* = 20], PKDL [*n* = 18], VL relapse [*n* = 3], and CL [*n* = 1]) as monotherapy. It was interesting that patients administered with L-AmB and MF combination therapy (*n* = 24) did not report fever and vomiting but had facial flushing, back pain, sweating, tachycardia, and cough more frequently (4%) as compared to monotherapy L-AmB (about 1%) patients. Surprisingly, the combination of L-AmB and PM did not result in ADRs, but the sample size of this group was small to make any conclusive assessments (see Table 2).

### Unexpected ADRs and SAEs

**Fig. 3:**
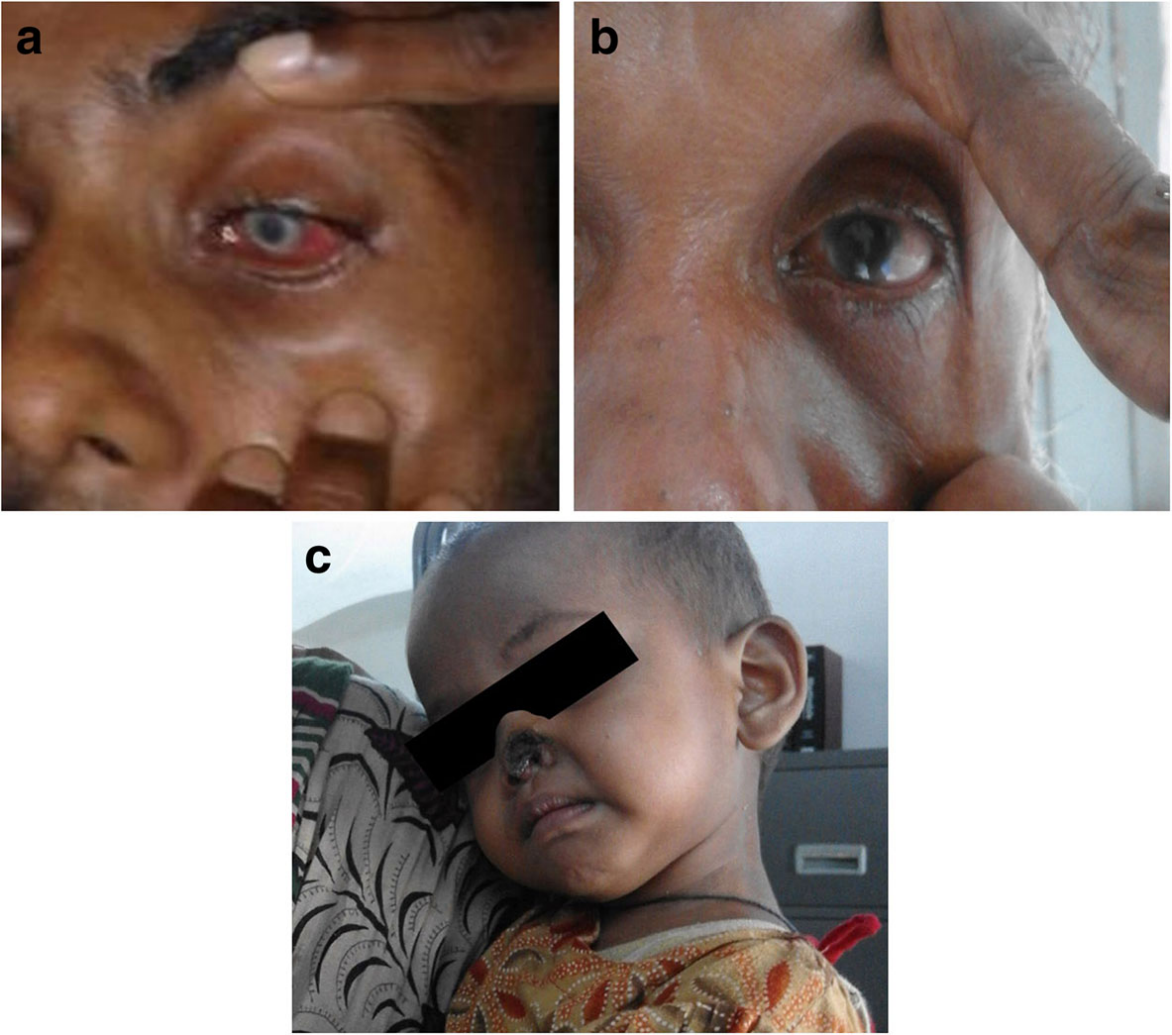
**a** Unexpected serious adverse event: Anular corneal ulcer. **b** Unexpected serious adverse event: Morren’s ulcer. **c** Unexpected serious adverse event: Avascular necrosis of the nasal alae

The focused VLPV activities found some SAEs that were not reported earlier. These were: annular corneal ulcer (see Fig. 3a), Mooren’s ulcer and peripheral ulcerative keratitis (see Fig. 3b), and necrosis of nasal alae (see Fig. 3c). The expert committee assessed ophthalmic SAEs as being possibly related to MF treatment of PKDL and necrosis of the nasal alae as being related to L-AmB treatment of VL.

Patients with eye complications started to feel eye problems in the second month of treatment with MF. The first two patients (with annular corneal ulcer and Mooren’s ulcer) followed the instruction of the VLPV team and sought medical care from the VLPV team when the eye complication began. However, the third patient continued treatment despite the eye problem and sought medical care from a private doctor. When there was no improvement, this patient finally came to the VLPV team. The patient was then referred to a specialized eye hospital in Dhaka. Subsequently, the patient has permanent disability with blindness in his affected eye. The 18-month-old child aged who had necrosis of the nasal alae was taken to a plastic surgeon who recommended surgical treatment when the child is of appropriate age. Fortunately, after a few weeks, natural healing of the affected part of the nose was observed.

### Deaths

Eight deaths were registered (four male and four female). Their ages ranged from 17 to 75 years. Six were new VL cases and two were VL relapses; all were treated with L-AmB monotherapy. The majority had a body mass index (BMI) < 16.5 and one patient had comorbidity with tuberculosis. The expert committee assessed three deaths as possibly being related to acute renal injury that occurred during treatment and two to possible myocardial infarction.

### Reporting to VigiFlow

All ADR reports were forwarded to the ADRM Cell and are already in VigiFlow.

## Discussion

The VLPV program has ensured the reporting of all expected and unexpected ADRs through the participation of all treatment centers. The initial challenge of the program was the low participation of treatment centers in terms of VLPV reporting. In addition to that some patients were treated in tertiary hospitals, which were not under the surveillance of the NKEP and did not have a VLPV team. These resulted in underreporting of 261 VLPV reports. The underreporting from the treatment centers was overcome through repeated refresher training. However to ensure VLPV reporting by the tertiary hospital the establishment of VLPV team in those should be taken care of by NKEP. Repeated training and refresher training at treatment centers is very important for conducting pharmacovigilance activities. This is reflected in the number of treatment centers reporting cases, which increased from 38% (10) to 100% (26) after the third refresher training. Therefore, the program should consider conducting periodic training of pharmacovigilance teams at the UHC to keep pharmacovigilance reporting functional. As focused pharmacovigilance has proven to be successful in the Bangladeshi context, similar initiatives may be taken up by other member countries conducting VL elimination programs, as well as other countries where leishmaniasis is prevalent.

During the study period, 42 types of expected ADRs were reported, with 26% of patients experiencing at least one episode of an ADR. The frequency of expected ADRs observed in this study is lower than the frequency of ADRs observed in many other clinical trials [9–11, 21–23]. In this study, 608 new VL cases were included, who were treated with L-AmB, MF, L-AmB and MF, L-AmB and PM, or MF and PM. The study conducted by Mondal et al. reported that 20% of new VL patients treated with single-dose AmBisome® experienced ADRs [11]. Another study reported that 27% of new VL patients treated with L-AmB experienced at least one episode of ADR [10]. Therefore the frequency (31%) of ADRs observed in new VL patients included in this study, who were treated with L-AmB, is not surprising compared to the abovementioned two studies. According to the national guidelines of Bangladesh, the first-line drug for new VL cases is L-AmB, therefore very few new VL patients are treated with MF.

According to this study, 17% of patients treated with MF experienced ADRs. Among the 12 new VL patients treated with MF, 17% experienced vomiting, 17% experienced nausea, and 8% experienced anorexia. A phase IV trial on MF [9], which was conducted during 2006 to 2007 in Bangladesh, reported that vomiting and diarrhea occurred in 25 and 8% of patients, respectively, among a total of 997 VL patients. A similar phase IV trial was conducted in Bihar, India, in which 401 ADRs were observed among 646 VL patients treated with MF [23]. Compared to the earlier studies, the observed frequency of ADRs related to MF treatment of new VL patients was lower in this study.

Unlike MF and L-AmB, PM is administered as part of combination therapy for treating VL patients in Bangladesh [10]. A phase IV trial on PM monotherapy to treat VL patients reported that 65% of patients experienced ADRs [21]. Another study reported 30 and 36% ADRs with L-AmB + PM and MF + PM combinations respectively for treatment of VL [10]. It is surprising that no ADR was observed among VL patients included in this study who were treated with PM combination therapy. The above evidence infers that PM combination therapy is safer than PM monotherapy to treat VL patients.

Unlike new VL cases, the percentage of observed ADRs after L-AmB monotherapy (26%) in PKDL patients was higher than that after MF monotherapy (16%). Furthermore, 20% of PKDL patients treated with MF and L-AmB combination therapy experienced ADRs. A study performed on PKDL patients to investigate the efficacy of MF reported that 50% of the patients experienced ADRs [22]. This anomaly can be attributed to the higher dose of MF administered to patients in that study. The above evidence substantiates the use of MF as the drug of choice for PKDL treatment.

The observed percentage of ADRs among relapse VL cases was 26%, which is comparable to the observed ADRs among new VL patients. The episodes of ADRs among relapse VL patients might have been reduced if combination therapy was used, since no ADRs were observed among relapse VL patients who were treated with combination therapy. Last but not the least, the treatment regimens for treatment failure and CL cases should be monitored carefully as significant proportions of these cases experienced ADRs. Fortunately most of the ADRs were not severe and did not require referral to the higher treatment centers. Nevertheless, careful monitoring of patients for the detection of ADRs and their management should be performed to ensure the safety and quality of life of patients while treating with antileishmanial drugs.

One of this study’s striking findings is the unexpected SAEs, particularly a case of an 18-month-old baby with L-AmB induced avascular necrosis of the nasal alae and another four cases of ophthalmic complications after taking MF for varying durations for treatment of PKDL. Maruf et al. suggested that the child who experienced avascular necrosis might have poor clearance of intravenous lipid emulsion, resulting in increased free fatty acid plasma levels following liposomal amphotericin infusion, which can lead to capillary embolism and vascular occlusion [24]. Pharmacovigilance activity shows that there is a risk of using L-AmB in children aged less than 5 years, and MF is also not a drug of choice for this age group. Therefore to treat children with VL, PM might be considered. The other patients with ocular SAEs developed those complications mostly during the fifth week of MF monotherapy, which probably occurred as a result of the accumulation of phospholipids in various ocular structures due to phospholipidosis, resulting in dry eye syndrome (Proggananda Nath et al., personal communication). It is worth mentioning that VLPV activity facilitated early detection and management of SAEs in most of these cases, which saved three patients from losing their sight. Therefore in the future, proper health education must be delivered to all PKDL patients treated with MF monotherapy, and these patients should also be advised to stop treatment immediately in the event of such complications and seek help from a VLPV team. The program should take initiative to update the health professionals regarding this particular ADR. Health professionals can then sensitize their patients on this complication while providing MF treatment.

In addition to ADRs, the focused pharmacovigilance activity enabled the identification of eight deaths related to antileishmanial management. Further investigation explored the plausible reasons of these deaths, with an association found between severe malnutrition and coinfection. Therefore the program should take initiative for separate management approaches while treating subsets of patients with low BMIs and comorbidity.

The major limitation of this study was underreporting of expected and unexpected ADRs. Treatment-related data could not be collected for 20% of the patients during the period from December 2014 to December 2016. Collecting data from all patients would have increased the chance of acquiring more expected and unexpected ADRs. Initially pharmacovigilance activity was not established in all VL treatment centers. Therefore ADR data for all patients treated by antileishmanial drugs were not reported during the study period. Another limitation is that the UMC grading system was not used for ADRs. However, ADR grading according to Common Toxicity Criteria guidelines will be performed upon the completion of the VLPV activity [25]. Further, no laboratory data are available for some subclinical ADRs related to antileishmanial drugs. This was not possible because national guideline recommends that additional laboratory tests should be performed only in presence of clinically significant sign and symptoms.

## Conclusions

Focused VLPV is feasible in Bangladesh’s public health system. In a public health program, when drugs are available only in public health facilities, hospital monitoring and active follow-up surveillance of treated patients are the most useful methods for conducting pharmacovigilance activities. VLPV activity is also useful for early detection of SAEs, and their proper management will ensure the safety of patients. Hence, VLPV should be continued and integrated into the public health system as a routine activity.

## Additional files

Additional file 1: Multilingual abstracts in the five official working languages of the United Nations. (PDF 834 kb)

Additional file 2: Operational Plan for Pharmacovigilance Activities for VL in Bangladesh. (PDF 558 kb)

## Abbreviations

ABD: Amphotericin B deoxycholate
ADR: Adverse drug reaction
ADRM: Adverse drug reaction monitoring
BMI: Body mass index
CDC: Center of Disease Control
CL: Cutaneous leishmaniasis
DGDA: Directorate General of Drug Administration
DGHS: Directorate General of Health Services
GoB: Government of Bangladesh
icddr,b: International Centre for Diarrhoeal Disease Research, Bangladesh
ISC: Indian subcontinent
L-AmB: Liposomal amphotericin B
MF: Miltefosine
NKEP: National Kala-azar Elimination Programme
PKDL: Post-kala-azar dermal leishmaniasis
PM: Paromomycin
SAE: Serious adverse event
SKKRC: Surya Kanta Kala-azar Research Center
UHC: Upazila health complex
VL: Visceral leishmaniasis
VLPV: Pharmacovigilance for visceral leishmaniasis
WHO: World Health Organization
WHO-UMC: World Health Organization Uppsala Monitoring Centre
