# Supplementary material for: Using focused pharmacovigilance for ensuring patient safety against antileishmanial drugs in Bangladesh’s National Kala-azar Elimination Programme
Source: Infect Dis Poverty. 2018 Aug 13;7:80. doi: 10.1186/s40249-018-0461-0 (PMC6088425; doi:10.1186/s40249-018-0461-0)
Supplement: Supplementary file 1 — Multilingual abstracts in the five official working languages of the United Nations. (PDF 834 kb) [file 40249_2018_461_MOESM1_ESM.pdf]

Translation of the abstract into the five official working languages of the United Nations

استخدام الصيدلة الاحتراسية المركزة (سلامة الدواء) لضمان سلامة المرضى ضد العقاقير المضادة لليشمانيات في برنامج بنغلاديش الوطني للقضاء على الداء الأسود (كالازار)

ساخوات حسين، أمريش كومار، أختار حسين، مهشن، أبهيجيت شارما، أكتر حسين، فارون شارما، رشيد الحق، شمسوزامان، شوميك معروف، براكاش غوش، فيفيك أهوجا، دينيش موندال

ملخص

الخلفية: التأثيرات الضارة للأدوية المضادة لليشمانيات يمكن أن تؤثر على نوعية حياة المرضى ومواظبتهم على استخدام العلاج ضد داء الليشمانيات الحشوي وداء الليشمانيات الجلدي التالي للكالازار. في بنغلاديش، هناك 26 مركز علاجي يعالج حالات داء الليشمانيات القادمة من 100مقاطعة فرعية متوطنة/موبوءة تتبع لـ26 مقاطعة (تتضمن داء الليشمانيات الحشوي، داء الليشمانيات الجلدي التالي للكالازار، فشل العلاج، انتكاسات (عودة) داء الليشمانيات الحشوي وداء الليشمانيات الجلدي). هدفت هذه الدراسة إلى دراسة جدوى استخدام الصيدلة الاحتراسية المركزة (سلامة الدواء) ضد داء الليشمانيات الحشوي في برنامج بنغلاديش الوطني للقضاء على الداء الأسود (كالازار) وذلك للكشف المبكر عن التفاعلات الدوائية الضارة المتوقعة وغير المتوقعة ومنعها.

الطرق: هذا النشاط مستمر منذ ديسمبر/كانون الأول 2014. يشمل نطاق النشاط المستشفى العام الثانوي أو مجمعات الصحة في 100مقاطعة فرعية ومركز"سوريا كانتا" لأبحاث الكلازار في منطقة مايمنسينغ وهو مركز متخصص لعلاج الحالات المعقدة من داء الليشمانيات الحشوي وداء الليشمانيات الجلدي التالي للكالازار. حدد مركز مكافحة الأمراض السارية التابع للمديرية العامة للخدمات الصحية 25 مجمعاً من أصل100مجمع للصحة في المقاطعات الفرعية إضافة لمركز"سوريا كانتا" لأبحاث الكلازار(الإجمالي 26) كمراكز للعلاج اعتماداً على موقعها الجغرافي المناسب. تم تنفيذ هذا من أجل معالجة أفضل لحالات داء الليشمانيات الحشوي بواسطة عقار "أمفوتيرسين بي" الشحمي (AmBisome®) وذلك لضمان راحة المريض والاستخدام الصحيح لهذا الدواء باهظ الثمن المتبرع به. تم إنشاء لجنة خبراء في الصيدلة الاحتراسية المركزة (سلامة الدواء) ضد داء الليشمانيات الحشوي وفريق داء الليشمانيات الحشوي بمجمعات الصحة في المقاطعات الفرعية، حيث تم تطوير دليل التشغيل ونماذج لتقارير الصيدلة الاحتراسية (سلامة الدواء)، وجرى تدريب وإعادة تدريب العاملين الصحيين على مستوى مجمعات الصحة في المقاطعات الفرعية وعلى المستوى المركزي، وتم تحليل معلومات مجمعة مثل بيانات المريض بما في ذلك الديموغرافيات (الخصائص السكانية)، وتاريخ (سجل) العلاج والاستجابة، والأحداث السلبية/العكسية. يتضمن هذا التقرير معلومات عن الفترة من ديسمبر/كانون الأول 2014 حتى ديسمبر/كانون الأول 2016.

النتائج: في الفترة من ديسمبر/كانون الأول 2014 حتى ديسمبر/كانون الأول 2016، تم علاج 1327 مريضاً من داء الليشمانيات، استفاد 1066 منهم أي (80%) من الصيدلة الاحتراسية المركزة (سلامة الدواء) ضد داء الليشمانيات الحشوي. حيث كانت 57%، 33%، 9%، 1% من الحالات في صفوفهم على التوالي: داء الليشمانيات الحشوي (حالات جديدة)، داء الليشمانيات الجلدي التالي للكالازار، داء الليشمانيات الحشوي (انتكاس)، حالات أخرى. غالباً ما كان يستخدم عقار "أمفوتيرسين بي" الشحمي (82%) لعلاج الحالات، يليه ميلتيفوسين (20%) و باروموميسين(3%). ومن أصل 1066 مريض، عانى 26% منهم من حالات التفاعلات الدوائية الضارة. التفاعلات الدوائية الضارة الأكثر شيوعاً هي الحمى (17%، 1066/176)، يليها القيء (5%، 1066/51). ولوحظ وجود13حدث سلبي/عكسي خطير (ثمانية وفيات وخمسة أحداث سلبية/عكسية خطيرة غير متوقعة). قيَّمت لجنة الخبراء أن ثلاثة من الوفيات وكافة الأحداث السلبية/العكسية الخطيرة غير المتوقعة ربما تتعلق بالعلاج. ومن بين الأحداث السلبية/العكسية الخطيرة غير المتوقعة الخمسة، كان هناك أربع حالات لمضاعفات عينية محرضة بـعقار "ميلتيفوسين"، أما الحالة الخامسة الأخيرة فكانت نخر إنعدام الأوعية في جناحي الأنف المحرض بـ عقار "أمفوتيرسين بي" الشحمي(AmBisome®). أدخلت المديرية العامة لإدارة الدواء التفاعلات الدوائية الضارة إلى قاعدة بيانات"فيجي فلو"VigiFlow في مركز أوبسالا للمراقبة التابع لمنظمة الصحة العالمية.

الاستنتاجات: وجدت هذه الدراسة أن الصيدلة الاحتراسية (سلامة الدواء) ضد داء الليشمانيات الحشوي من خلال البرنامج الوطني للقضاء على الداء الأسود (كالازار) هي أمر ممكن، ويجب أن تستمر كنشاط روتيني في نظام الصحة العامة في بنغلاديش لضمان سلامة المرضى من العقاقير المضادة لليشمانيات.

Translated from English version into Arabic by Ammar Alhosain, proofread by Free Bird, through

TRANSLATORS WITHOUT BORDERS

在孟加拉国的国家黑热病消除计划中采用监测重点药物不良反应以确保抗利什曼药物的安全使用

Sakhawat Hossain, Amresh Kumar, Akhtar Hossain, Mahshin, Abhijit Sharma, Akter Hossain, Varun Sharma, Rashidul Haque, Shamsuzzaman, Shomik Maruf, Prakash Ghosh, Vivek Ahuja and Dinesh Mondal

摘要

引言：抗利什曼病药物的不良反应会影响患者生活质量，及对内脏利什曼病（VL）和黑热病后皮肤利什曼病（PKDL）治疗的依从性。在孟加拉国，有 26 个治疗中心管理着来自 26 个地区 100 个流行区（亚区）的利什曼病患者（包括 VL、PKDL、治疗失败、VL 复发和皮肤利什曼病患者）。本研究旨在研究在孟加拉国

国家黑热病消除计划中使用针对 VL 的药物不良反应监测（VLPV）可行性，以便对预期的和意外的药物不良反应(ADRs)进行早期发现和预防。
方法：本项目始于 2014 年 12 月，涉及迈门辛 100 个流行区的二级公立医院或地区医疗中心（UHC），以及专门管理复杂 VL 和 PKDL 患者的 Surya Kanta 黑热病研究中心(SKKRC)。隶属于卫生服务部（DGHS）的疾病预防控制中心（CDC）根据合适的地理位置指定了 25 个 UHC 和 SKKRC 作为治疗中心。从而，能更好地使用两性霉素 B 脂质体（AmBisome®）治疗 VL 病例，以确保患者方便和正确使用这种昂贵的捐赠药物。目前建立了 VLPV 专家委员会和 UHC VLPV 团队，制定了操作手册和药物不良反应监测报告程序。在 UHC 和中央层面对卫生人员进行培训和在职复修培训。收集患者数据，包括人口统计数据、病史和不良反应，分析了不良事件。本报告包括 2014 年 12 月至 2016 年 12 月的相关信息。
结果：2014 年 12 月至 2016 年 12 月，共有 1327 例利什曼病患者接受治疗，其中 1066 例（80％）可适用 VLPV。患者中 57％、33％、9％和 1％分别是新 VL、PKDL、VL 复发和其他病例。两性霉素 B 脂质体被广泛（82％）用于治疗患者，其次是米替福新（20％）和巴龙霉素（3％）。在 1066 例患者中，26％出现 ADR。最常见的 ADR 是发热（17％，176/1066）、其次是呕吐（5％，51/1066）。观察到 13 个严重不良事件（SAEs）（8 例死亡和 5 例意外的 SAEs）。专家委员会评估说，其中 3 例死亡和所有意外的 SAE 可能与治疗有关。5 例意外的 SAE 中，4 例是米替福新诱发的眼部并发症，另一例为 AmBisome®诱发的鼻翼缺血性坏死。药物管理总局将 ADR 纳入世界卫生组织乌普萨拉监测中心(WHO-UMC) VigiFlow 数据库。
结论：本研究发现在孟加拉国国家黑热病消除计划中使用 VLPV 是可行的，应作为孟加拉国公共卫生系统的常规活动继续进行，以确保患者使用抗利什曼病药物的安全性。

Translated from English version into Chinese by Peng Song, edited by Pin Yang

CHINESE CENTER FOR DISEASE CONTROL AND PREVENTION
NATIONAL INSTITUTE OF PARASITIC DISEASES

Utilisation de la pharmacovigilance ciblée pour assurer la sécurité des patients vis-à-vis des médicaments antileishmaniens dans le cadre du Programme national d’élimination du kala-azar au Bangladesh

Sakhawat Hossain, Amresh Kumar, Akhtar Hossain, Mahshin, Abhijit Sharma, Akter Hossain, Varun Sharma, Rashidul Haque, Shamsuzzaman, Shomik Maruf, Prakash Ghosh, Vivek Ahuja et Dinesh Mondal

Résumé
Contexte: les effets indésirables des médicaments antileishmaniens peuvent affecter la qualité de vie des patients et l’observance du traitement contre la leishmaniose viscérale (LV) et la leishmaniose dermique post-kala-azar (LDPKA). Il existe au Bangladesh 26 centres de traitement qui prennent en charge les cas de leishmaniose venus de 100 *upazilas* (sous-districts) d’endémie dans 26 districts (cas de LV et de LDPKA, d’échec de traitement et de récidive de leishmaniose viscérale ou cutanée). La présente étude visait à étudier la faisabilité de l’utilisation d’une pharmacovigilance ciblée pour la LV (PV-LV) dans le cadre du Programme national d’élimination du kala-azar au Bangladesh (NKEP), afin de détecter précocement et de prévenir les effets indésirables prévisibles et inattendus des médicaments (EIM).
Méthodes: Cette activité est en place depuis décembre 2014. La zone d’activité comprend les hôpitaux publics secondaires (complexes médicaux des *upazilas*, UHC) de 100 sous-districts et le Centre de recherche sur le kala-azar Surya Kanta (SKKRC) dans le district de Mymensingh, qui est spécialisé dans la gestion des cas compliqués de VL et de LDPKA. Le service de lutte contre les maladies transmissibles (CDC) de la Direction générale des services de santé (DGHS) a désigné 25 des 100 UHC et le SKKRC (soit 26 centres au total) pour servir de centres de traitement, sur la base de leur situation géographique. Le but était de mieux prendre en charge les cas de LV avec de l’amphotéricine B liposomale (AmBisome®), afin de faciliter le traitement pour les patients et d’utiliser à bon escient ce coûteux médicament, obtenu par des dons. Un comité d’experts en PV-LV et une équipe de PV-LV dans chaque UHC ont été mis en place. Un manuel opératoire et des formulaires de signalement de pharmacovigilance ont été élaborés, la formation initiale et continue des personnels de santé a été organisée dans les UHC et au niveau central, et les informations recueillies (données des patients, notamment données démographiques, historique du traitement et réponse, les événements indésirables) ont été analysées. Le présent rapport couvre la période de janvier 2014 à décembre 2016.

Résultats: Entre décembre 2014 et décembre 2016, 1327 patients souffrant de leishmaniose ont été traités et 1066 (80 %) ont été suivis par la PV-LV. Parmi ceux-ci, 57 % étaient de nouveaux cas de VL, 33 % de nouveaux cas de LDPKA, 9 % des récidives de VL et 1 % étaient dans un autre cas. La plupart des patients (82 %) ont été traités avec de l'amphotéricine B liposomale, suivie de la miltéfosine (20 %) et de la paromomycine (3 %) Sur les 1066 patients, 26 % ont eu des effets indésirables. L'EIM le plus fréquent était la fièvre (17 %, 176 sur 1066), suivie des vomissements (5 %, 51 sur 1066). Treize cas d'effets indésirables graves (huit décès et cinq EIG inattendus) ont été observés. Le comité d'experts a estimé que trois des décès et tous les EIG inattendus pouvaient être liés au traitement. Sur les cinq effets indésirables graves inattendus, quatre étaient des complications ophtalmiques induites par la miltéfosine et le dernier une nécrose avasculaire des ailes du nez induite par l'AmBisome®. La Direction générale de l'administration des médicaments a versé les EIM dans la base de données VigiFlow, au Centre de surveillance de l'Organisation mondiale de la santé (WHO-UMC) à Uppsala.
Conclusions: Cette étude a révélé qu'une PV-LV était possible dans le cadre du NKEP est faisable et devrait être intégrée comme une activité de routine dans le système de santé publique du Bangladesh afin d'assurer la sécurité des patients qui prennent des médicaments antileishmaniens.

Translated from English version into French by Emmanuelle Hardan, proofread by Suzanne Assenat, through

TRANSLATORS WITHOUT BORDERS

Осуществление фармокологического надзора в целях обеспечения безопасности пациентов при применении лекарств от лейшманиоза в рамках Национальной программы Бангладеш по борьбе с висцеральным лейшманиозом (кала-азар)

Сахават Хоссейн, Амреш Кумар, Ахтар Хоссейн, Махшин, Абхиджит Шарма, Актер Хоссейн, Варун Шарма, Рашидул Хак, Шамсуззаман, Шомик Маруф, Пракаш Гхош, Вивек Ахуджа и Динеш Мондал

Аннотация
Исходная информация: Неблагоприятное воздействие лекарственных препаратов от лейшманиоза способно влиять на качество жизни пациентов и на приверженность к лечению от висцерального лейшманиоза (ВЛ) и пост-кала-азар кожного лейшманиоза (ПККЛ). В Бангладеш действуют 26 центров по лечению лейшманиоза, вспышки которого зафиксированы в 100 упазилах (подокругах) 26 округов (включая случаи ВЛ, ПККЛ, неэффективность лечения, а также рецидивы ВЛ и случаи кожного лейшманиоза). Целью настоящего исследования являлся анализ целесообразности применения целенаправленного фармакологического надзора при лечении ВЛ (ФНВЛ) в рамках Национальной программы Бангладеш по борьбе с кала-азар для своевременного выявления и профилактики ожидаемых и неожиданных неблагоприятных воздействий лекарств (НДЛ).
Методы: Исследование проводится с декабря 2014 г. Место проведения исследования включает непрофильную государственную больницу или подокружной центр здравоохранения (ПЦЗ) в ста подокругах, а также центр изучения кала-азар Сурья Канта (SKKRC) в округе Мименсингх, специализированный центр лечения осложнённых случаев ВЛ и ПККЛ. Служба санитарно-эпидемиологического надзора за инфекционными заболеваниями (КИЗ) Генерального директората по здравоохранению (ГДЗ) назначила центрами лечения двадцать пять из ста ПЦЗ и SKKRC (всего 26) в зависимости от удобства их географического положения. Данная мера была направлена на повышение качества лечения ВЛ при помощи липосомального амфотерицина В (AmBisome®) с целью обеспечения удобного и надлежащего использования пациентами данного дорогостоящего препарата, предоставленного в качестве гуманитарной помощи. Была создана экспертная комиссия ВЛФН и команда по ФНВЛ при ПЦЗ, разработана инструкция по эксплуатации и формы отчётов фармокологического контроля, проведено обучение и переподготовка медицинского персонала в ПЦЗ и на центральном уровне, собрана информация, в частности, сведения о пациентах, включая демографические данные, историю лечения и реакцию на лечение, а также проанализированы случаи неблагоприятного действия препаратов. Настоящий отчёт содержит информацию за период с декабря 2014 года по декабрь 2016 года.
Результаты:За период с декабря 2014 года по декабрь 2016 года прошли лечение 1327 пациентов и 1066 пациентов (80 %) смогли пройти ВЛФН. Из них 57 %, 33 %, 9 % и 1 % составили соответственно: новый

случай ВЛ, ПККЛ, рецидив ВЛ и прочее. Для лечения в большинстве случаев применялся липосомальный амфотерицин B (82 %), реже — милтефозин (20 %) и паромомицин (3 %). Из числа 1066 пациентов у 26% был зафиксирован НДЛ. Наиболее распространённые симптомы НДЛ включали повышенную температуру (17%, 176/1066), реже — рвоту (5%, 51/1066). Были зафиксированы тринадцать случаев серьёзных нежелательных явлений (СНЯ): восемь смертей и пять непредвиденных случаев СНЯ. Эспекртная комисиия установила, что три случая смерти и все случаи СНЯ, предположительно, были спровоцированы лечением. Четыре из пяти случаев СНЯ включали офтальмологические осложнения, вызванные применением милтефозина, а также один — аваскулярный некроз носовой полости — был вызван применением AmBisome®. Генеральный директорат по применению лекарственных препаратов внёс зафиксированные случаи НДЛ в базу данных VigiFlow Центра мониторинга Всемирной организации здравоохранения в Упсале.
Выводы: В ходе настоящего исследования было установлено, что ФНВЛ в рамках Национальной программы Бангладеш (NKEP) является целесообразным и подлежит постоянному применению в системе здравоохранения Бангладеш для обеспечения безопасности пациентов при применении лекарств от лейшманиоза.

Translated from English version into Russian by DreamTranslator, proofread by Liudmila Tomanek, through

TRANSLATORS WITHOUT BORDERS

Utilización de la farmacovigilancia enfocada para garantizar la seguridad del paciente ante los fármacos contra la leishmaniasis en el Programa Nacional de Eliminación de Kala-azar de Bangladesh

Sakhawat Hossain, Amresh Kumar, Akhtar Hossain, Mahshin, Abhijit Sharma, Akter Hossain, Varun Sharma, Rashidul Haque, Shamsuzzaman, Shomik Maruf, Prakash Ghosh, Vivek Ahuja y Dinesh Mondal

Resumen

Contexto: Los efectos adversos de los fármacos contra la leishmaniasis pueden afectar la calidad de vida del paciente y el cumplimiento con su terapia para la leishmaniasis visceral (LV) y la leishmaniasis dérmica post-kala-azar (LDPK). En Bangladesh, hay 26 centros de atención que tratan casos de leishmaniasis provenientes de 100 upazilas (subdistritos) endémicas de 26 distritos (incluyendo casos de LV, LDPK, fracaso del tratamiento y recurrencia de LV y leishmaniasis cutánea). Este estudio tiene como propósito investigar la viabilidad de la utilización de la farmacovigilancia enfocada para la LV (VLPV, por sus siglas en inglés) en el Programa Nacional de Eliminación de Kala-azar de Bangladesh para la detección temprana y la prevención de esperadas e inesperadas reacciones adversas a los fármacos (ADR, por sus siglas en inglés).
Métodos: Esta actividad se ha llevado a cabo desde diciembre de 2014. La zona de actividad incluye el hospital público secundario o el complejo de salud de la upazila (UHC, por sus siglas en inglés) en 100 subdistritos y el Centro de Investigación de Kala-azar Surya Kanta (SKKRC, por sus siglas en inglés) en el distrito Mymensingh, un centro especializado en el tratamiento de casos complicados de LV y LDPK. El Control de Enfermedades Transmisibles (CDC, por sus siglas en inglés) de la Dirección General de Servicios de Salud (DGHS, por sus siglas en inglés) asignó a 25 de 100 UHC y al SKKRC (por un total de 26) como centros de tratamiento según su ubicación geográfica. Esto se implementó para mejorar el tratamiento de casos de LV con anfotericina B liposomal (AmBisome®) para asegurar la conveniencia del paciente y el uso apropiado de este costoso fármaco donado. Se estableció un comité de expertos en la VLPV y un grupo de VLPV de UHC, se desarrolló un manual operacional y un formulario para gestionar informes de farmacovigilancia, la capacitación y la capacitación de repaso del personal de salud tuvo lugar en los UHC y a nivel central; se analizó la información recopilada de los pacientes, como los datos demográficos, los antecedentes de tratamiento y su respuesta, y los acontecimientos adversos. Este informe incluye información sobre el período de tiempo entre diciembre de 2014 y diciembre de 2016.
Resultados: De diciembre de 2014 a diciembre de 2016, se trataron 1327 pacientes con leishmaniasis y 1066 (el 80 %) fueron elegibles para la VLPV. De ellos, el 58 %, 33 %, 9 % y 1 % fueron casos nuevos de leishmaniasis visceral, leishmaniasis dérmica post-kala-azar, recurrencia de leishmaniasis visceral y otros casos, respectivamente. Se utilizó mayormente la anfotericina B liposomal (82 %) para el tratamiento de los casos, seguida por la miltefosina (20 %) y la paromomicina (3 %). De los 1066 pacientes, el 26 % tuvo una ADR. La reacción adversa más frecuente

fue fiebre (17 %, 176/1066), seguida por vómitos (5 %, 51/1066). Se observaron trece acontecimientos adversos graves (SAE, por sus siglas en inglés) (ocho muertes y cinco SAE inesperados). El comité experto concluyó que tres de las muertes y todos los SAE inesperados estuvieron posiblemente vinculados con el tratamiento. De los cinco SAE inesperados, cuatro se debieron a complicaciones oftálmicas causadas por la miltefosina y el otro se debió a una necrosis avascular de las alas nasales causada por AmBisome ®. La Dirección General de Administración de Fármacos ingresó las ADR en la base de datos VigiFlow del Centro de Seguimiento Uppsala de la Organización Mundial de la Salud (WHO-UMC, por sus siglas en inglés).
Conclusión: La investigación determinó que la VLPV a través del Programa Nacional de Eliminación de Kala-azar (NKEP, por sus siglas en inglés) es viable y debe continuar como un procedimiento rutinario en el sistema público de salud de Bangladesh para garantizar la seguridad del paciente ante los fármacos contra la leishmaniasis.

Translated from English version into Spanish by Jannette Gonzalez, proofread by Marigli, through

TRANSLATORS
WITHOUT BORDERS
