# Supplementary material for: Using focused pharmacovigilance for ensuring patient safety against antileishmanial drugs in Bangladesh’s National Kala-azar Elimination Programme
Source: Infect Dis Poverty. 2018 Aug 13;7:80. doi: 10.1186/s40249-018-0461-0 (PMC6088425; doi:10.1186/s40249-018-0461-0)
Supplement: Supplementary file 2 — Operational Plan for Pharmacovigilance Activities for VL in Bangladesh. (PDF 558 kb) [file 40249_2018_461_MOESM2_ESM.pdf]

Supplement 1

OPERATIONAL PLAN FOR PHARMACOVIGILANCE ACTIVITIES FOR VISCERAL LEISHMANIASIS IN BANGLADESH

Document Version	1.00			
Effective Date	15 SEPTEMBER 2014			
Approved by	Prof. Be-Nazir Ahmed	Mr. Salim Barami		Dr. Vivek Ahuja
Signatures				
Institution	CDC	DGDA	Icddr,b	PATH

TABLE OF CONTENTS

1 Acronyms and AbbrevIATIONS 3

2 Background 4

3 Purpose 5

4 Scope 5

5 Procedures 5

5.1 Roles and Responsibilities 5

5.1.1 Healthcare Professional (HCP) 5

5.1.2 Focal Person at Kala-azar Treatment Centers: 6

5.1.3 Nursing Staff 6

5.1.4 Responsible Person at the CDC 6

5.1.5 icddr,b 7

5.1.6 DGDA 7

5.2 Information to be collected 8

5.3 Reporting Format and Procedures 8

5.4 Timelines/Frequency of Reporting 9

5.4.1 Fatal Cases 9

5.4.2 Non-fatal AEs 9

5.5 Analysis of the Reported Adverse Drug Reactions information 9

5.5.1 Expert committee 10

6 Flowchart 1

1 ACRONYMS AND ABBREVIATIONS

ADRAC	Adverse Drug Reactions Advisory Committee
AE	Adverse Events
CDC	Centre of Disease Control
DGDA	Directorate General of Drug Administration
GoB	Government of Bangladesh
HCP	Health Care Professionals
ICH	International Conference on Harmonisation
LAMB	Liposomal amphotericin B
PDS	Pharmacoepidemiology and Drug Safety
PKDL	Post Kala-azar Dermal Leishmaniasis
PV	Pharmacovigilane
SAE	Serious Adverse Events
UHC	Upazilla Health Complex
VL	Visceral Leishmaniasis (VL);
WHO	World Health Organization
WHO-UMC	World Health Organization – Uppsal Monitoring Centre

2 BACKGROUND

The National Guideline on Kala-azar and PKDL case management in Bangladesh 2013 has been updated and enforced.[1]This operational plan recommends various regimens with the use of following drugs for treatment of Kala-azar:

1) Liposomal amphotericin B (LAMB)
2) Amphotericin B
3) Miltefosine
4) Paromomycin

All these drugs are used in Bangladesh for prescription to VL and or PKDL patients under the National VL Elimination program.

This operational plan also recommends that pharmacovigilance[1] data on the safety of above drugs and their regimen should be systematically collected from the various health facilities in the country. Pharmacovigilance is the science and activities relating to the detection, assessment, understanding and prevention of adverse effects or any other drug-related problems[2].

Adverse effects due to drugs used for treatment of Kala-azar can significantly affect the quality of life and adherence to therapy.[3] An effective model of pharmacovigilance with particular focus to Kala-azar will ensure that the patients of this disease are able to accrue the maximum therapeutic benefit by early detection and prevention of adverse effects of these drugs. This will be possible by following a systematic system for surveillance, collection, detection, assessment and monitoring of adverse effects.

This document details the pharmacovigilance activities to be carried out by the health care professionals involved in the management of patients of Kala-azar as well as Post Kala-azar Dermal Leishmaniasis (PKDL). The pharmacovigilance program will act in harmony with the Kala-azar elimination national program to collect data regarding adverse drug reactions and

[1] Pharmacovigilance isThe science and activities relating to the detection, assessment, understanding and prevention of adverse effects or any other drug-related problems.

also assess and evaluate these reactions for causality and benefit-risk profile and, in future may provide the basis for risk mitigation plans. This activity will not only strengthen the capacity of the Kala Azar Elimination Program but also the National Pharmacovigilance Program of the Government of Bangladesh.

3 PURPOSE

This Pharmacovigilance activity is designed to improve patient outcomes associated with the use of medicines for VL. The primary objective of this document is to define the procedures for pharmacovigilance of anti-leishmanial drugs used in Bangladesh for establishing the practical working methods for reporting adverse events (AE) by health care professionals (HCP).

4 SCOPE

This operational plan applies to all Healthcare Professionals (HCP) engaged with diagnosis or treatment of Visceral Leishmaniasis (VL) and Post Kala azar Dermal Leishmaniasis (PKDL). It should also apply to those dispensing drugs used for treatment of these disease conditions; provide nursing care to such patients or other personnel directly involved in the collection and analysis of adverse drug reaction data for these drugs. This document should be used as an operational plan by the personnel listed above. All activities will be conducted in accordance with this plan and the national guidelines for treatment of VL/PKDL. The most recent approved version of this guidance document will take precedence over any other version.

5 Procedures

All reports of adverse events following administration of a drug or drugs for treatment of Kala-azar will be collected and reported. Details of this procedure are as follows:

5.1 Roles and Responsibilities

5.1.1 Healthcare Professional (HCP)

All Healthcare Professionals (HCP) engaged with the following activities have a role to play in the AE reporting:

- Diagnosis or treatment of Visceral Leishmaniasis (VL) and Post Kala azar Dermal Leishmaniasis (PKDL), or
- Dispensing of drugs used for treatment of these disease conditions, or
- Providing nursing care to such patients, or
- Personnel directly involved in the collection and analysis of AE data for these drugs

5.1.2 Focal Person at Kala-azar Treatment Centers:

- A Focal Person will be identified by Head of the treatment center at each centre. Their contact details will be made available to the Centre of Disease Control (CDC), GoB
- The identified focal persons will undergo a targeted training on the operational plan
- Focal person will be responsible for filling the AE form. The treating physician will sign the form in the end, before sending it to CDC.
- In case the focal person is the treating physician he/she will sign the AE form(s).

5.1.3 Nursing Staff

- Nursing staff should promptly report to the doctor any suspected adverse drug reaction coming to their notice.

5.1.4 Responsible Person at the CDC

- Provide list of the Upazilla Health Complex and name of the participants to be trained and wo will be responsible for reporting from the their respective UHCs
- Facilitate and support training of Medical Doctors, Nurses and reporting personnel from Upazilla Health Complex (UHC)
- Collect through e-mail/Fax/Courier/Web based system the Adverse Event Report prepared by Upazilla Health Complex (UHC)
- Verify for completeness of the form and get updates from UHC in need
- Approve the form for data entry and send it to DGDA and icddr,b
- Conduct the data entry and share it with icddr,b periodically.

- Select the members for Causality Assessment Committee and chair the committee.
- Support and facilitate the meeting of the Causality Assessment Committee and present the analyzed data to the committee

5.1.5 icddr,b

- Maintain liaison with PDS, CDC and DGDA and contribute to preparation of form and manual
- Obtain all required approval from competent authorities of icddr,b for this activity
- Support training of Medical Doctors, Nurses and reporting personnel from Upazilla Health Complex (UHC)
- Prepare database program for data entry following the indicators in the reporting form
- Provide human resource to support CDC for performing data entry and to support the VL surveillance team
- Arrange the meeting of the Causality Assessment Committee and present the analyzed data to the committee
- Share the report of the committee with CDC and DGDA

5.1.6 DGDA

- Support training of Medical Doctors, Nurses and reporting personnel from Upazilla Health Complex (UHC)
- Receive soft copy of the AE form from CDC
- Enter data in Vigiflow
- Present data to National Adverse Drug Reaction Committee
- Send recommendations from ADRAC to CDC-Kala Azar Elimination Program and DGDA for regulatory action

5.2 Information to be collected

All patients should be monitored regularly for signs and symptoms indicative of an AE. Any suspected AE in a patient undergoing treatment of Kala-azar or PKDL should be collected and recorded promptly. For timelines on reporting of the AEs to the CDC and Directorate General of Drug Administration (DGDA), the following should be kept in mind while reporting:

- Cases of death and life threatening events/incidents must be given highest priority and reported on an expedited basis
- Both known and unknown AEs should be reported.

5.3 Reporting Format and Procedures

The Adverse Drug Reaction Reporting form (AE Reporting Form, Appendix 1) must be filled for an individual patient. Separate such forms should be filled for different patients. Responsibilities for filling this form are as follows:

- Focal person will be responsible for filling the AE form.
- The doctor/treating physician will sign the form in the end, before sending it to CDC
- The narrative for every new AE should be written on an additional sheet of paper and appended to the AE reporting form
- Each AE form must have the following mandatory information:
    - An identifiable patient
    - A suspect medicinal product
    - An identifiable reporting source
    - At least one adverse drug reaction
- As much information about the patient's history and the AE should be captured for more effective analysis. Address for Sending the AE Report
- Filled and signed form should be sent as hard copy via web based, or by fax, or by soft copy as attachment via email data transfer to the CDC. The address to send is given below:

Send to: Ms. Fahima Yeasmin

Address: CDC, DGHS, GoB,
Mohakhali, Dhaka-1212

Email ID: lira_urp@yahoo.com

Phone: +880-2- 8816412

Fax: +880-2-8813875

5.4 Timelines/Frequency of Reporting

5.4.1 Fatal Cases

- Fatal cases suspected as a result of AEs due to anti Kala-azar drug(s) should be reported within seven calendar days of knowledge of such event

5.4.2 Non-fatal AEs

- All other AEs irrespective of seriousness or expectedness should be reported on a monthly basis. Following will apply for monthly reporting:
  - AE reporting forms should be sent by 15th of every month, and will contain data from first to last day of the previous month
  - If an AE report is received on 28th of a month, then the initial report will be sent to CDC within that month's reporting cycle. CDC physician will revert to the district /zonal centers on inadequate data in the form and if it is not enough to assess causality.

5.5 Analysis of the Reported Adverse Drug Reactions information

The AE data collected at CDC will be shared with the Directorate general of Drug Administration, , Government of Bangladesh.

The following steps will be executed at the CDC by the responsible person after receipt of the AE information:

- An acknowledgement of the receipt will be sent back to the sender

- A unique identification number will be allotted to each patient. Receipt of any follow up information will be added under this unique number. The same patient will not be given a new number
- Data will be quality checked, medically evaluated and causality will be assessed according to the WHO-UMC causality assessment algorithm (Appendix 2)
- The data will be analyzed for signals/alerts

Based on the data analysis & processing, appropriate recommendations & risk mitigation plans will be generated & communicated to various stakeholders

5.5.1 Expert committee

An Expert Committee at the CDC will be formed to recommend actions based on the data analysis.

The Expert Committee will consist of the following members:

- o CDC director: Chair
- o Academia: Pharmacology/pharmacy – 2 persons as member
- o One DGDA representative as member
- o Two Senior physician (Kala-azar expert) as member
- o One International PV Expert as member as on need basis

Role of the Expert Committee will be as follows:

- Review the data/information: half yearly, or as required
- Make recommendation to Kala-azar elimination program

6 Flowchart

Flow Chart for Operational plan for Pharmacovigilance Activities for Visceral Leishmaniasis in Bangladesh

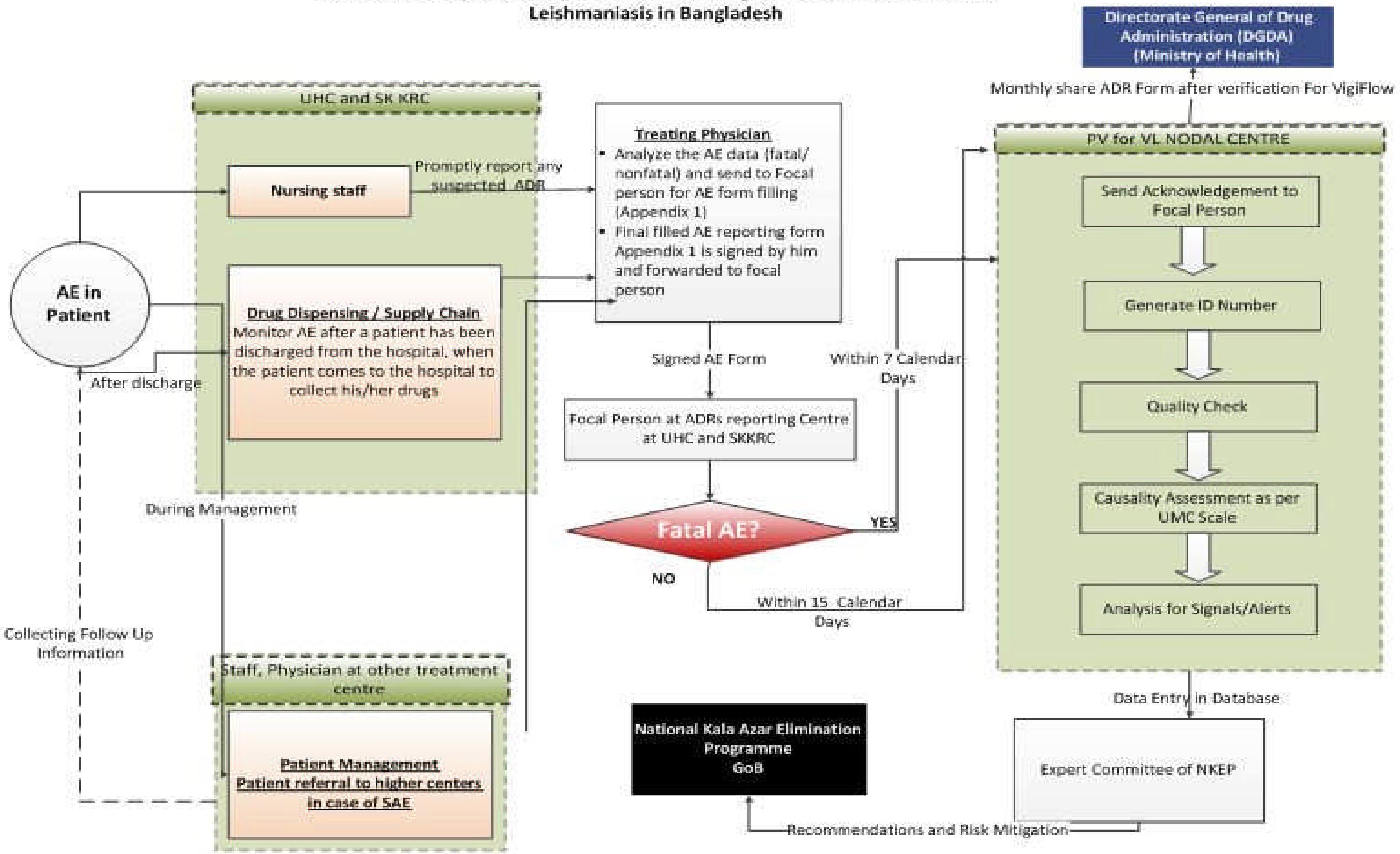

Appendix 1: Adverse Drug Reaction Reporting Form

Suspected Adverse Event reporting Form

Identities of reporter, patient, institution, and product trade name(s) will remain confidential

Suspected Adverse Drug Reaction or Adverse Event Reporting Form

Identities of reporter, patient, institution, and product trade name(s) will remain confidential

ADR report number____ (For office use only)

Date received____

**A.PATIENT AND HOSPITAL INFORMATION**		
Name of health facility:		
Patient name :		Registration no:
Patient address:	District:<br>Union/ Moholla/Street no.:	Upazila:<br>Village/Holding no./Para:
Contact number:		
Age (Years) __________ Weight (kg) ___________ Height(cm) ________ Gender ◯ Male ◯ Female		
Pregnant : ◯ Yes ◯ No ◯ Unknown ◯ Not applicable		
Don't enter Full name in excel sheet, Please enter initial		

**B.HISTORY OF VL THERAPY**					
Treatment received	◯ Combo Therapy			◯ Mono Therapy	
Drug received	☐ Liposomal Amphotericin B	☐ Miltefosine	☐ Paromomycin	☐ Non-Liposomal Amphotericin B	☐ Sodium Stibo Gluconate
Trade name					
Drug dose					
Drug start date*					
Drug start time**					
Drug stop date *					
Drug stop time**					

*Date format: DD/MM/YYYY; **Time format: HH:MM AM/PM

**C. SUSPECTED ADVERSE EVENT INFORMATION**		
**Type of event**<br>☐ Adverse drug reaction<br>☐ Product quality problem<br>☐ Medication error<br>☐ Other:_______________	**Suspected product**<br>Trade name _________________Generic name____________________________<br>Indication____<br>Dose [strength, unit]______________________ Dosage form_________________<br>Frequency____<br>Batch/Lot number __________________Manufacturer______________________	
**Describe event including relevant tests and laboratory results:**		
Date & Time the event started<br>___________________________<br>(DD/MM/YYYY; HH:MM AM/PM)	Date & Time the event was reported<br>_____________________________<br>(DD/MM/YYYY; HH:MM AM/PM)	Date & Time the event stopped<br>____________________________<br>(DD/MM/YYYY; HH:MM AM/PM)
**Was the adverse event treated?**<br>**If yes, please specify**	◯ Yes ◯ No	

**Action taken after the Adverse Drug Reaction or Adverse Event**<br>◯ Dose stopped<br>◯ Dose reduced<br>◯ No action taken	**Did reaction subside after stopping/reducing the dose of the suspected Product?**<br>◯ Yes ◯ No ◯ Not applicable<br>**Did reaction appear after reintroducing the suspected product?**<br>◯ Yes ◯ No ◯ Not applicable

**Seriousness of the adverse event:**	**Outcomes of the adverse event:**
◯ Not serious<br>◯ Hospitalization or prolongation of hospitalization<br>◯ Disability or permanent damage<br>◯ Congenital anomaly/birth defect<br>◯ Life threatening<br>◯ Other serious<br>◯ Death	◯ Recovered<br>◯ Recovered/resolved with sequela<br>◯ Not recovered<br>◯ Unknown<br>◯ Fatal (date of death: ________________________)
Other relevant history (including pre-existing medical conditions, allergies, pregnancy, smoking, alcohol use, liver or kidney problems, hypersensitivity, history of ADRs, etc.):	

D. OTHER CONCOMITANT PRODUCT INFORMATION

	Product 1	Product 2	Product 3	Product 4
Trade name				
Generic name				
Indication				
Dosage form				
Route				
Dose				
Frequency				
Date started*				
Time started**				
Date stopped*				
Time stopped**				

*Date format: DD/MM/YYYY; ** Time format: HH:MM AM/PM

E. REPORTER INFORMATION

Name ____Designation____________________________________

Email address____

Mobile phone ____Land phone____________________________________

Signature ____Date of submission___________________________

List of Adverse Events

General
- [ ] Fever
- [ ] Chills
- [ ] Rigor
- [ ] Peripheral edema
- [ ] Asthenia
- [ ] Malaise
- [ ] Fatigue
- [ ] Lethargy
- [ ] Sweating
- [ ] Facial flushing
- [ ] Rash
- [ ] Vertigo

Respiratory
- [ ] Dyspnea
- [ ] Cough
- [ ] Lung disorder
- [ ] Pleural effusion
- [ ] Rhinitis
- [ ] Hypoxia
- [ ] Hyperventilation
- [ ] Asthma
- [ ] Atelectasis
- [ ] Hemoptysis
- [ ] Hiccup
- [ ] Flu-like symptom
- [ ] Lung edema
- [ ] Pharyngitis
- [ ] Pneumonia
- [ ] Respiratory insufficiency
- [ ] Sinusitis

Cardio-vascular
- [ ] Hypotension
- [ ] Tachycardia
- [ ] Hypertension
- [ ] Chest pain
- [ ] Phlebitis
- [ ] Arrhythmia
- [ ] Atrial fibrillation
- [ ] Bradycardia
- [ ] Cardiac arrest
- [ ] Cardiomegaly
- [ ] Hemorrhage
- [ ] Postural hypotension
- [ ] ECG abnormalities
- [ ] Venous pain
- [ ] Venous thrombosis

Gastro-intestinal
- [ ] Dyspepsia
- [ ] Hematemesis
- [ ] Anorexia
- [ ] Diarrhea
- [ ] Vomiting
- [ ] Abdominal pain
- [ ] Nausea
- [ ] Abdominal distention
- [ ] Constipation
- [ ] Dysphagia
- [ ] Flatulence
- [ ] Melena
- [ ] Peptic ulcer
- [ ] Dry mouth
- [ ] Epigastric pain

Hepato-biliary
- [ ] Jaundice
- [ ] Hyperbilirubinemia
- [ ] Hepatomegaly
- [ ] Veno-occlusive disorder
- [ ] Rise in hepatic enzymes
- [ ] Pancreatitis

Renal
- [ ] Nephrotoxicity
- [ ] Renal failure
- [ ] Serum creatinine elevation

Genito-urinary
- [ ] Hematuria
- [ ] Dysurea
- [ ] Urinary incontinence
- [ ] Vaginal hemorrhage
- [ ] Testicular pain
- [ ] Testicular swelling
- [ ] Scrotal pain
- [ ] Decreased ejaculate volume
- [ ] Absent ejaculation

Nervous
- [ ] Insomnia
- [ ] Confusion
- [ ] Peripheral neuropathy
- [ ] Motion sickness
- [ ] Headache
- [ ] Dizziness
- [ ] Somnolence
- [ ] Paresthesia
- [ ] Ototoxicity

Hematologic
- [ ] Anemia
- [ ] Leucopenia
- [ ] Thrombocytopenia
- [ ] Gum bleeding
- [ ] Bleeding from nose

Muscolo-skeletal
- [ ] Back pain
- [ ] Bone pain
- [ ] Dystonia
- [ ] Myalgia
- [ ] Rhabdomyolysis
- [ ] Arthralgia
- [ ] Tetany
- [ ] Sub sternal pain

Metabolic
- [ ] Hypokalemia
- [ ] Hypomagnesemia
- [ ] Hyperglycemia
- [ ] Hypocalcemia
- [ ] Hypovolemia
- [ ] Hyponatremia
- [ ] Hypernatremia

Dermatologic
- [ ] Urticaria
- [ ] Pruritus
- [ ] Cellulitis
- [ ] Erythema
- [ ] Pyoderma
- [ ] Rash
- [ ] Urticaria
- [ ] Steven-johnson syndrome

Immunological
- [ ] Lymphangitis

Local
- [ ] Abscess
- [ ] Ear puritus
- [ ] Injection site swelling
- [ ] Injection site pain

Occular
- [ ] Conjunctivitis
- [ ] Dry eye
- [ ] Hemorrhage

Hypersensitivity
- [ ] Allergic reaction

Psychiatric
- [ ] Anxiety
- [ ] Depression
- [ ] Hallucination
- [ ] Nervousness
- [ ] Abnormal thoughts

Others
- [ ] ....
- [ ] ....
- [ ] ....

- [ ] ***Death***
- [ ] ***Birth defect***

Appendix 2. WHO-UMC causality assessment algorithm

Causality term	Assessment criteria*
**Certain**	• **Event or laboratory test abnormality, with plausible time relationship to drug intake**<br>• **Cannot be explained by disease or other drugs**<br>• **Response to withdrawal plausible (pharmacologically, pathologically)**<br>• **Event definitive pharmacologically or phenomenologically (i.e. an objective and specific medical disorder or a recognized pharmacological phenomenon)**<br>• **Rechallenge satisfactory, if necessary**
**Probable/Likely**	• **Event or laboratory test abnormality, with reasonable time relationship to drug intake**<br>• **Unlikely to be attributed to disease or other drugs**<br>• **Response to withdrawal clinically reasonable**<br>• **Re-challenge not required**
**Possible**	• **Event or laboratory test abnormality, with reasonable time relationship to drug intake**<br>• **Could also be explained by disease or other drugs**<br>• **Information on drug withdrawal may be lacking or unclear**
**Unlikely**	**Event or laboratory test abnormality, with a time to drug intake that makes a relationship improbable (but not impossible)**<br>• **Disease or other drugs provide plausible explanations**

**Conditional/ Unclassified**	• **Event or laboratory test abnormality**<br>• **More data for proper assessment needed, or**<br>• **Additional data under examination**
**Unassessable/ Unclassifiable**	• **Report suggesting an adverse reaction**<br>• **Cannot be judged because information is insufficient or contradictory**<br>• **Data cannot be supplemented or verified**

***All points should be reasonably complied with**

Appendix 3. Definitions

1. **Pharmacovigilance** *[The World Health Organization (2002)]*

The science and activities relating to the detection, assessment, understanding and prevention of adverse effects or any other drug-related problems. Pharmacovigilance is sometimes referred to as Drug Safety surveillance or Drug monitoring

Following are broadly the function of pharmacovigilance:

- Detection and study of adverse reactions
- Measurement of risk
- Measurement of effectiveness
- Benefit & harm evaluation
- Dissemination of information, education
- Early warning
- Rational and safe use of medicines

2. Health Care Professional (HCP) [ICH E2D]

An HCP is a medically-qualified person such as a physician, dentist, pharmacist, nurse, coroner, or as otherwise specified by local regulations.

3. Adverse Event (or Adverse Experience) *[ICH E2A]*

Any untoward medical occurrence in a patient or clinical investigation subject administered a pharmaceutical product and which does not necessarily have to have a causal relationship with this treatment.

4. Adverse Drug Reaction (AE) *[ICH E2A]*

Pre-approval:

All noxious and unintended responses to a medicinal product related to any dose.

-causal relationship cannot be ruled out.

Marketed products:

A response to a drug which is noxious and unintended and which occurs at doses normally used in man for prophylaxis, diagnosis, or therapy of disease or for modification of physiological function.

5. **Serious Adverse Event *[ICH E2A]***

An Adverse Event which:

- Results in death, OR
- Is life-threatening*, OR
- Requires inpatient hospitalization or prolongation of existing hospitalization, OR
- Results in persistent or significant disability/incapacity, OR
- Is a congenital anomaly/birth defect, OR
- Is medically significant

*Life threatening: If the patient was at substantial risk of dying at the time of the adverse event or it is suspected that the use or continued use of the product would result in the patient's death. D*oes not include an adverse event that, had it occurred in a more severe form, might have caused death*

In-patient Hospitalization: *Any new in-patient hospitalization or prolongation of existing hospitalization.*

If a patient comes in the emergency due to an adverse event, the event will be assessed as serious only if the patient gets in-patient hospitalized or the investigator feels it to be serious due to some other applicable seriousness criteria

Hospitalization (including hospitalization for an elective procedure) for a pre-existing condition (prior to study entry) which has not worsened does not constitute a serious adverse event.

Persistent or significant disability or incapacity: *If the adverse event resulted in a significant, persistent, or permanent change, impairment, damage or disruption in the patient's body function/structure, physical activities or quality of life.*

Congenital anomaly or birth defect: *If there is any congenital anomaly or birth defect in offspring of subjects or their partners taking the investigational medicinal product regardless of time of diagnosis.*

Medically significant: *Medical and scientific judgment should be exercised in other situations, such as important medical events that may not be immediately life-threatening or result in death or hospitalization but may jeopardize the patient or may require intervention to prevent one of the other outcomes listed in the definition earlier.*

E.g. Allergic bronchospasm requiring intensive treatment at home, convulsions that do not result in inpatient hospitalization

6. **Severity - of an Adverse Event *[ICH E2A]***

The term severe is used to describe the intensity of a specific event. The terms serious and severe are not synonymous.

- An event can be severe but may not be serious (e.g. severe headache). Similarly an event can be serious but may not be severe (e.g. mild myocardial infarction)

7. **Unexpected Adverse Drug Reaction/Adverse Event**

An adverse event, the nature, severity, specificity or outcome of which is not consistent with the applicable product information*

*Prescribing Information /Pack Insert/ Summary of Product Characteristics

8. **Relatedness – of an Adverse Event**

AE/SAE may be related or unrelated to the drug. This is done by Causality assessment, which is defined as:

“The evaluation of the likelihood that a medicine is the causative agent of an observed adverse event”

9. Signals/Alerts:
   Information that arises from one or multiple sources (including observations and experiments), that suggests a new potentially causal association, or a new aspect of a known association, between an intervention and an event or set of related

events, either adverse or beneficial, that is judged to be of sufficient likelihood to justify further action to verify. (ICH)

Refrences

[1] The National Kala Azar Elimination Guidelines for Bangladesh are under publication and final approval by the Department of Epidemiology and Disease Control, Government of Bangladesh.

[2] WHO PV Definition
[3] Cite publicatons which describe the known AEs of VL Drugs
